# Preoperative predictors for non-resectability in perihilar cholangiocarcinoma

**DOI:** 10.1186/s12957-024-03329-1

**Published:** 2024-02-07

**Authors:** Carlos Constantin Otto, Anna Mantas, Lara Rosaline Heij, Daniel Heise, Maxime Dewulf, Sven Arke Lang, Tom Florian Ulmer, Edgar Dahl, Philipp Bruners, Ulf Peter Neumann, Jan Bednarsch

**Affiliations:** 1 Department of Surgery and Transplantation, University Hospital Essen, Essen, Germany; 2 Department of Surgery and Transplantation, University Hospital RWTH Aachen, Aachen, Germany; 3 Department of Surgery, Maastricht University Medical Center (MUMC), Maastricht, Netherlands; 4 Institute of Pathology, University Hospital RWTH Aachen, Aachen, Germany; 5 Department of Radiology, University Hospital RWTH Aachen, Aachen, Germany

**Keywords:** Klatskin tumor, Non-resectability, Peritoneal neoplasms, Staging laparoscopy

## Abstract

**Introduction:**

Explorative laparotomy without subsequent curative-intent liver resection remains a major clinical problem in the treatment of perihilar cholangiocarcinoma (pCCA). Thus, we aimed to identify preoperative risk factors for non-resectability of pCCA patients.

**Material and methods:**

Patients undergoing surgical exploration between 2010 and 2022 were eligible for the analysis. Separate binary logistic regressions analyses were used to determine risk factors for non-resectability after explorative laparotomy due to technical (tumor extent, vessel infiltration) and oncological (peritoneal carcinomatosis, distant nodal or liver metastases)/liver function reasons.

**Results:**

This monocentric cohort comprised 318 patients with 209 (65.7%) being surgically resected and 109 (34.3%) being surgically explored [explorative laparotomy: 87 (27.4%), laparoscopic exploration: 22 (6.9%)]. The median age in the cohort was 69 years (range 60–75) and a majority had significant comorbidities with ASA-Score ≥ 3 (202/318, 63.5%). Statistically significant (*p* < 0.05) risk factors for non-resectability were age above 70 years (HR = 3.76, *p* = 0.003), portal vein embolization (PVE, HR = 5.73, *p* = 0.007), and arterial infiltration > 180° (HR = 8.05 *p* < 0.001) for technical non-resectability and PVE (HR = 4.67, *p* = 0.018), arterial infiltration > 180° (HR = 3.24, *p* = 0.015), and elevated CA 19–9 (HR = 3.2, *p* = 0.009) for oncological/liver-functional non-resectability.

**Conclusion:**

Advanced age, PVE, arterial infiltration, and elevated CA19-9 are major risk factors for non-resectability in pCCA. Preoperative assessment of those factors is crucial for better therapeutical pathways. Diagnostic laparoscopy, especially in high-risk situations, should be used to reduce the amount of explorative laparotomies without subsequent liver resection.

**Supplementary Information:**

The online version contains supplementary material available at 10.1186/s12957-024-03329-1.

## Introduction

Perihilar cholangiocarcinoma (pCCA) is the most common subtype of CCA, usually diagnosed in the advanced disease stage and mostly associated with poor oncological outcomes [1]. Liver resection remains the gold standard of therapy and the only option for long-term survival in patients with pCCA [2]. Over the last decades, surgical therapy has evolved from isolated resection of the extrahepatic bile duct to extended liver resection with vascular reconstructions as well as multivisceral resections resulting in increased resectability rates for patients with advanced pCCA [3–9]. Unfortunately, recurrence rates after curative intent surgery remain high [10]. Surgical margin, lymph node status, tumor differentiation as well as the involvement of vessels are the main prognostic factors for oncological outcomes after curative-intent surgery [11]. In case of irresectability due to cirrhosis or technical concerns, a small proportion of patients who are no candidates for resection might also be applicable to curative-intent therapy by transplantation after neoadjuvant therapy. Of note, the assessment for transplantation is strictly characterized by adherence to regulative protocols, e.g., the Mayo protocol [12]. However, most patients diagnosed with pCCA are not eligible for operative resection at the time of diagnosis due to distant metastasis or extensive vascular involvement at the liver hilum [1, 13, 14].

Despite the improvement of numerous preoperative imaging modalities, e.g., multiphase computer tomography (CT), magnetic resonance cholangiopancreatography (MRCP)/magnetic resonance imaging (MRI) and positron emission tomography (PET)–CT, a notable amount undergoes surgical exploration and is intraoperatively assessed as not being resectable due to surgical reasons (infiltration of vessels, etc.), impaired liver function or previously undetected peritoneal carcinomatosis and distant lymph node metastases [1, 13]. At this point, there is no staging system sufficiently predicting resectability in pCCA [15].

Surgical exploration is associated with significant cost, impaired quality of life, postoperative complications, unnecessary hospitalization, and a delay in systemic therapy. Therefore, we here aimed to investigate the role of surgical exploration in patients with pCCA and identify preoperative predictors for irresectability of patients with pCCA undergoing operative exploration in curative intent.

## Material and methods

### Patients

All consecutive patients with pCCA who underwent operative exploration with curative intent at the University Hospital RWTH Aachen (UH-RWTH) between 2010 and 2022 were included in this retrospective study. Patients diagnosed with intrahepatic cholangiocarcinoma involving the liver hilum pre- or postoperatively were not investigated in this study. This study was conducted in concordance with the requirements of the Institutional Review Board of the RWTH-Aachen University (EK 23–270) and the current version of the Declaration of Helsinki as well as the good clinical practice guidelines (ICH-GCP). The utilized data was collected retrospectively and saved in an institutional database.

### Staging and surgical procedure

All included patients preoperatively underwent a detailed preoperative clinical work-up as described previously [16, 17]. To classify tumor extent, endoscopic retrograde cholangiopancreatography (ERCP) and/or magnetic resonance cholangiopancreatography (MRCP) were conducted. Carbohydrate antigen 19–9 (CA19-9) was used as a tumor marker. The anatomical classification was described according to Bismuth-Corlette. Computed tomography (CT) with arterial and venous phases was the standard procedure to rule out distant metastases and assess a possible tumor invasion of perihilar vessels as well as the vascular anatomy of the liver. The preoperative imaging was not older than 4 weeks at the time of surgical exploration in every patient. Ultrasound-directed aspiration of suspicious locoregional lymph nodes was not carried out routinely as nodal metastases in regional nodes were not considered as a contraindication for surgical resection. Diagnostic laparoscopy was only carried out if there were clear diagnostic hints for the presence of peritoneal carcinomatosis. Unilateral stenting of the future remnant liver (FLR) was preferred, only in cases with refractory cholangitis, and persistent cholestasis bilateral stenting was performed. Generally, endoscopic biliary drainage (EBD) was preferred over percutaneous biliary drainage (PBD). Based on the results of preoperative FLR prediction, right portal vein embolization (PVE) was conducted in cases with insufficient FLR scheduled for right-sided hepatectomy 2 to 4 weeks before surgery. Generally, a FLR above 30% was considered as sufficient. In selected cases with borderline FLR or risk factors of impaired parenchyma quality, e.g., neoadjuvant therapy or chronic cholestasis, maximum liver function capacity (LiMAx) was carried out to assess metabolic liver function [18]. Neoadjuvant therapy was considered in cases that were preoperatively assessed as non-resectable or in cases with distant lymph node metastases. Furthermore, the classification of the American Society of Anesthesiologists (ASA) was used to objectify patients´ preoperative status. The decision for primary surgery was always made by an experienced hepatobiliary surgeon in accordance with the interdisciplinary tumor board. During surgical exploration, the resectability of the tumor was assessed by an experienced hepatobiliary surgeon. If resectability was ensured, liver resection with lymphadenectomy was carried out as previously described [19, 20]. Specimens were examined by an experienced pathologist both for intraoperative frozen sections as well as for the final tissue diagnosis.

### Reasons for non-resectability

During surgical exploration, the peritoneal cavity was meticulously examined for peritoneal carcinomatosis, liver metastases, or distant lymph node metastasis. If any of these observations were detected and verified by intraoperative frozen sections, liver resection was not pursued. Secondly, the quality of the liver parenchyma was assessed by inspection. If in contradiction to the preoperative evaluation the assumed liver function of the FLR was considered insufficient, the surgical procedure was also terminated. Also, in the case of an intraoperatively smaller FLR as initially planned e. g. due to the necessity to remove more liver volume or segments to achieve clear tumor margins, the procedure was terminated. Afterward, the liver hilum was also carefully explored by the attending surgeon. Here, technical reasons for non-resectability were assessed. The main reasons for technical non-resectability were tumor infiltration of major vessels without the possibility of reconstruction and unexpected tumor growth precluding an R0 resection. Furthermore, a larger tumor extent than expected resulting in a more extensive surgical procedure than planned (e.g., hepatopancreaticoduodenectomy or trisectionectomy instead of conventional left or right hepatectomy), was considered a contraindication if the patient’s physical fitness did not allow an escalation of the surgical procedure.

### Follow-up

After surgical resection, adjuvant therapy was recommended in cases of high-risk features (e.g., positive nodal status, R1 resection) between 2010 and 2017 in and after 2017 in any case according to the results of the BILCAP trial [21]. In patients who were considered not resectable, palliative systemic treatment was proposed to all patients during the study period.

### Statistical analysis

Categorical data are reported as absolute numbers and percentages. Continuous variables are displayed by median and interquartile range. The primary endpoint of our study was the identification of preoperative parameters associated with non-resectability for various reasons. Those parameters were identified by univariate and multivariable logistic regression analysis between patients being successfully resected and patients undergoing exploration without resection. Only statistically significant parameters (*p* < 0.05) in univariable analysis were subsequently analyzed with multivariable binary logistic regressions using backward elimination. Separate analyses were conducted for patients being non-resectable due to oncological/liver function and technical reasons. The secondary endpoint of the study was the comparison of cancer-specific survival (CSS) between resected and non-resected patients. CSS was defined as the time between the date of operation and the date of last contact (if the patient was still alive) or the date of tumor-related death. Survival curves were generated by the Kaplan–Meier method and compared with the log-rank test. Median follow-up was assessed using the reverse Kaplan–Meier method. Statistical significance was set at *p* ≤ 0.05. Confidence level is set to 95%. Analyses were performed with SPSS Statistics 24 (IBM Corp., Armonk, NY, USA).

## Results

### Patient cohort

The cohort consisted of 318 patients who underwent surgical exploration for pCCA in curative intent between 2010 and 2022 at our institution. The overall cohort was based on 206 male (64.8%) and 112 female patients (35.2%) with a median age of 69 years (range 60–75). Most of the patients had significant comorbidity with ASA-Score ≥ 3 (202/318, 63.5%). Bismuth type IV represented the most frequent pCCA type (96/318, 30.2%), followed by Bismuth Type IIIa (86/318, 27%) and Bismuth Type IIIb (69/318, 21.7%). A subset of 18 patients (5.7%) underwent neoadjuvant chemotherapy. Tumor infiltration of portal vein (145/318, 45.7%) or hepatic artery (83/318, 26.2%) was widely observed in the preoperative radiologic imaging.

The cohort was divided into groups for further analysis: The majority of patients successfully underwent oncological resection of the tumor (“resected cohort”, 209/318, 65.7%), while the remaining patients underwent either conventional or laparoscopic exploration and were considered not resectable (“overall exploration cohort”, 109/318, 34.3%). The overall exploration group was further used for survival analysis to determine the oncological role of non-resectability. The subgroup of patients who were explored by explorative laparotomy was further used for regression analysis to determine the role of unnecessary surgical laparotomies (explorative laparotomy group (87/318, 27.4%). Patients undergoing surgical exploration without resection who were later resected after chemotherapy or other optimization methods were not allocated to the exploration but resection cohort.

The most frequent reasons for non-resectability in the overall cohort were peritoneal carcinomatosis (46/109, 42.2%), vascular infiltration (20/109, 18.3%), a larger resection extent than expected (14/109, 12.8%), distant lymph node metastases (12/109, 11.0%), impaired liver function/liver cirrhosis (9/109, 8.3%), and liver metastases (5/109, 4.6%).

Detailed demographic and clinicopathological statistics of the relevant subcohorts are displayed in Table 1.

**Table 1:** Patients’ characteristics

Demographics	Resected cohort (*n* = 209)	Overall exploration (*n* = 109)	Exploration without laparoscopy (*n* = 87)
Gender, m/f (%)	139 (66.5)/70 (33.5)	67 (61.5)/42 (38.5)	51 (58.6)/36 (41.4)
Age (years)	68 (58–74)	70 (62–76)	72 (64–76)
BMI (kg/m^2^)	25.3 (22.8–28.4)	25.5 (22.9–28.8)	25.4 (22.6–28.9)
Bismuth classification, *n* (%)			
I	11 (5.3)	7 (6.4)	7 (8)
II	28 (13.4)	20 (18.3)	16 (18.4)
IIIa	61 (29.2)	25 (22.9)	18 (20.7)
IIIb	51 (24.4)	18 (16.5)	15 (17.2)
IV	57 (27.3)	39 (35.8)	31 (35.6)
Neoadjuvant therapy, *n* (%)	10 (4.8)	8 (7.3)	5 (5.7)
Portal vein embolization, *n* (%)	74 (35.4)	18 (16.5)	14 (16.1)
ASA, *n* (%)			
I	8 (3.8)	4 (3.7)	2 (2.3)
II	71 (34)	33 (30.3)	25 (28.7)
III	116 (55.5)	66 (60.6)	55 (63.2)
IV	14 (6.7)	6 (5.5)	5 (5.7)
V	0	0	0
Preoperative cholangitis, *n* (%)	67 (32.1)	27 (24.8)	31 (35.6)
Preoperative EBD, *n* (%)	150 (71.8)	80 (73.4)	63 (72.4)
Preoperative PBD, *n* (%)	52 (24.9)	27 (24.8)	25 (28.7)
Portal vein infiltration > 180°, *n* (%)			
None	117 (56)	55 (50.5)	43 (49.4)
Main	3 (1.4)	23 (21.1)	19 (21.8)
Bifurcation	27 (12.9)	0	0
Right	22 (10.5)	9 (8.3)	8 (9.2)
Left	38 (18.2)	22 (20.2)	17 (19.5)
Right and left	1 (0.5)	0	0
Arterial infiltration > 180°, *n* (%)			
None	169 (80.9)	65 (59.6)	51 (58.6)
Main	0	15 (13.8)	12 (13.8)
Bifurcation	0	0	0
Right	34 (16.3)	25 (22.9)	21 (24.1)
Left	3 (1.4)	4 (3.7)	3 (3.4)
Right and left	2 (1)	0	0
Lobar atrophy,* n* (%)			
None	146 (69.9)	78 (71.6)	61 (70.1)
Right	9 (4.3)	2 (1.8)	2 (2.3)
Left	53 (25.4)	29 (26.6)	24 (27.6)
sFLR (%)	0.57 (0.39–0.72)	0.56 (0.44–0.7)	0.55 (0.43–0.67)
Clinical chemistry			
Albumin (g/dl)	3.8 (3.4–4.1)	3.7 (3.2–4.2)	3.7 (3.2–4.1)
AST (U/l)	45 (34–84)	52 (31–79)	48 (30–79)
ALT (U/l)	58 (35–111)	55 (32–100)	54 (28–101)
GGT (U/l)	403 (188–758)	394 (182–739)	393 (170–733)
Total bilirubin (mg/dl)	1.1 (0.6–2.8)	1.3 (0.8–3.2)	1.4 (0.8–3.2)
Platelet count (/nl)	295 (228–389)	278 (215–355)	281 (240–354)
Alkaline phosphatase (U/l)	266 (158–423)	251 (158–457)	247 (157–441)
Prothrombin time (%)	96 (84–105)	92 (77–102)	92 (75–102)
INR	1.03 (0.97–1.11)	1.05 (0.97–1.16)	1.05 (0.97–1.19)
Hemoglobin (g/dl)	12.2 (11–13.3)	12.3 (10.8–13.5)	12.4 (10.9–13.4)
CRP (mg/l)	12 (6–35.8)	18.9 (7.2–45.1)	19.4 (7–43)
CA 19–9 (U/ml)	99 (34–335)	293 (78–1387)	231 (79–1135)
Operative data			
Operative time (minutes)	450 (379–511)	120 (118.5–122.5)	120 (120–136)
Operative procedure, *n* (%)			
Limited bile duct resection	8 (3.8)	n.a	n.a
Right hepatectomy	26 (12.4)	n.a	n.a
Left hepatectomy	28 (13.4)	n.a	n.a
Mesohepatectomy	2 (1)	n.a	n.a
Extended right hepatectomy	42 (20.1)	n.a	n.a
Extended left hepatectomy	53 (25.4)	n.a	n.a
Right trisectionectomy	26 (12.4)	n.a	n.a
Left trisectionectomy	9 (4.3)	n.a	n.a
Hepatoduodenoectomy	13 (6.2)	n.a	n.a
ALPPS	0	n.a	n.a
Reasons for irresectability			
Liver function/Liver cirrhosis	n.a	9 (8.3)	5 (5.7)
Distant lymph nodes	n.a	12 (11)	12 (13.8)
Liver metastases	n.a	5 (4.6)	2 (2.3)
Peritoneal carcinomatosis	n.a	46 (42.2)	33 (37.9)
Vascular infiltration without	n.a	20 (18.3)	20 (23)
Possibility of reconstruction			
Resection larger than expected	n.a	14 (12.8)	13 (14.9)
Others	n.a	3 (2.8)	2 (2.3)
Portal vein reconstruction	152 (72.7)	n.a	n.a
Arterial reconstruction	16 (7.7)	n.a	n.a
Intraoperative PRBC	104 (49.8)	0	0
Intraoperative FFP	113 (54.1)	0	0
Pathological examination			
R1 resection, *n* (%)	40 (19.1)	n.a	n.a
pT category, *n* (%)		n.a	n.a
1	15 (7.2)	n.a	n.a
2	116 (55.5)	n.a	n.a
3	51 (24.4)	n.a	n.a
4	19 (9.1)	n.a	n.a
pN category			
N0	116 (55.5)	n.a	n.a
N1	92 (44)	n.a	n.a
Tumor grading, *n* (%)			
G1	8 (3.8)	n.a	n.a
G2	138 (66)	n.a	n.a
G3	49 (23.4)	n.a	n.a
G4	1 (0.5)	n.a	n.a
MVI, *n* (%)	62 (29.7)	n.a	n.a
LVI, *n* (%)	46 (22)	n.a	n.a
PNI, *n* (%)	147 (70.3)	n.a	n.a
Postoperative data			
Intensive care, days	2 (1–5)	0	0
Hospitalization, days	19 (12–35)	9 (5–21)	12 (6–22)
Postoperative complications, *n* (%)			
No complications	35 (16.7)	66 (60.6)	49 (56.3)
Clavien-Dindo I	12 (5.7)	9 (8.3)	8 (9.2)
Clavien-Dindo II	43 (20.1)	6 (5.5)	4 (4.6)
Clavien-Dindo IIIa	36 (17.2)	14 (12.8)	12 (13.8)
Clavien-Dindo IIIb	34 (16.3)	3 (2.8)	3 (3.4)
Clavien-Dindo Iva	11 (5.3)	2 (1.8)	2 (2.3)
Clavien-Dindo Ivb	7 (3.3)	0	0
Clavien-Dindo V	32 (15.3)	9 (8.3)	9 (10.3)
Oncologic data^a^			
Adjuvant therapy	57 (27.3)	n.a	n.a
Median CSS, months (95% CI)	32 (20-44)	6 (4–8)	n.a

Data presented as median and interquartile range if not noted otherwise

*ALT* Alanine aminotransferase, *ASA* American Society of Anesthesiologists classification, *AST* Aspartate aminotransferase, *BMI* Body mass index, *CCI* Comprehensive complication index, *CSS* Cancer-specific survival, *EBD* Endoscopic biliary drainage, *FFP* Fresh frozen plasma, *pCCA* Perihilar cholangiocarcinoma, *GGT* Gamma-glutamyltransferase, *INR* International normalized ratio, *LVI* Lympho-vascular invasion, *MVI* Microvascular invasion, *PBD* Percutaneous biliary drainage, *PNI* Perineural invasion, *PRBC* Packed red blood cells

^a^Oncologic data with the exclusion of perioperative mortality

### Logistic regression analyses for explorative laparotomy

As we aimed to primarily investigate unnecessary laparotomies, patients who were determined as not resectable by laparoscopy were excluded from the logistic regression analysis. We assessed risk factors for non-resectability due to technical reasons (vascular infiltration without the possibility of reconstruction or resection larger than expected). Therefore, those patients were investigated together with the resected cohort using univariate binary logistic regressions for explorative laparotomy as described in the given literature [22]. Parameters with a *p* value < 0.05 were defined as statistically relevant. In here, relevant risk factors were age above 70 years (hazard ratio (HR) = 2.43, Confidence interval (CI):1.15–5.16, *p* = 0.019), preoperative portal vein embolization (PVE, HR = 5.48, CI1.62–18.57, *p* = 0.001) and arterial infiltration of more than 180° (HR = 5.2, CI 2.41–11.22, *p* < 0.001). Those variables were further included in a multivariable logistic regression model to assess statistical independence. This model defined age above 70 years (HR = 3.76, CI 1.56–9.08, *p* = 0.003), PVE (HR = 5.73, CI 1.61–20.38, *p* = 0.007) and arterial infiltration > 180° (HR = 8.05, CI 3.32–19.53, *p* < 0.001) as independent predictors of non-resectability (Table 2).

**Table 2:** Logistic regression of preoperative parameters for non-resectability due to technical reasons

	Univariate analysis		Multivariate analysis	
	HR (95% CI)	*P* value	HR (95% CI)	*P* value
Demographics				
Sex (male = 1)		0.1		
Age (≤ 70 years = 1)	2.43 (1.15–5.16)	**0.019**	3.76 (1.56–9.08)	**0.003**
BMI (≤ 25 kg/m^2^ = 1)		0.561		
Bismuth type (I/II = 1)		0.087		
Neoadjuvant therapy (no = 1)		0.636		
Preoperative MR-Imaging (no = 1)		0.666		
PVE (no = 1)	5.48 (1.62–18.57)	**0.001**	5.73 (1.61–20.38)	**0.007**
ASA (I/II = 1)		0.401		
Preoperative cholangitis (no = 1)		0.84		
EBD (no = 1)		0.909		
PBD (no = 1)		0.315		
Portal vein infiltration > 180° (no = 1)		0.139		
Arterial infiltration > 180° (no = 1)	5.2 (2.41–11.22)	** < 0.001**	8.05 (3.32–19.53)	** < 0.001**
Lobar atrophy (no = 1)		0.765		
sFLR (≤ 40% = 1)		0.468		
Clinical chemistry				
Albumin (≤ 35 g/l = 1)		0.353		
AST (≤ 50 U/l = 1)		0.649		
ALT (≤ 50 U/l = 1)		0.315		
GGT (≤ 400 U/l = 1)		0.263		
Bilirubin (≤ 1 mg/dl = 1)		0.921		
Alkaline phosphatase (≤ 250 U/l = 1)		0.439		
Platelet count (≤ 300/nl = 1)		0.462		
INR (≤ 1 = 1)		0.364		
Hemoglobin (≤ 12 g/dl = 1)		0.819		
CRP, mg/l (≤ 10 mg/l = 1)		0.694		
CA 19–9, U/ml (≤ 250 U/ml = 1)		0.673		

Various parameters are associated with non-resectability. Statistically significant *p* values are presented in brackets

*ALT* Alanine aminotransferase, *ASA* American Society of Anesthesiologists classification, *AST* Aspartate aminotransferase, *BMI* Body mass index, *CRP* C-reactive protein, *EBD* Endoscopic biliary drainage, *GGT* Gamma-glutamyltransferase, *INR* International normalized ratio, *PBD* Percutaneous biliary drainage, *PVE* Portal vein embolization

A similar approach was used to identify risk factors for non-resectability due to oncological and liver function reasons. Cases with intraoperative diagnosed distant lymph nodes, liver metastases, or peritoneal carcinomatosis were added for oncological reasons. Non-resectability due to liver function was defined as an intraoperative diagnosis of cirrhosis. The combination of oncological and liver function reasons was chosen as these features might be also assessable by diagnostic laparoscopy. Hereby, PVE (HR = 2.62, CI 1.21–5.67, *p* = 0.009), arterial infiltration > 180° (HR = 2.29, CI 1.78–4.48, *p* = 0.017), and CA 19–9 > 250 U/ml (HR = 3.91, CI 1.71–8.93, *p* = 0.001) were significant in univariate logistic regression. Those parameters were further evaluated in multivariable logistic regression. In this analysis, PVE (HR = 4.67, CI 1.31–16.69, *p* = 0.018), arterial infiltration > 180° (HR = 3.24, CI:1.26–8.31, *p* = 0.015) and CA 19–9 > 250 U/ml (HR = 3.2, CI 1.33–7.69, *p* = 0.009) showed an independent association for non-resectability due to oncological and liver function reasons (Table 3).

**Table 3:** Logistic regression of preoperative parameters for non-resectability due to oncological reasons/liver function

	Univariate analysis		Multivariate analysis	
	HR (95% CI)	*P* value	HR (95% CI)	*P* value
Demographics				
Sex (male = 1)		0.503		
Age (≤ 70 years = 1)		0.223		
BMI (≤ 25 kg/m^2^ = 1)		0.976		
Bismuth type (I/II = 1)		0.454		
Neoadjuvant therapy (no = 1)		0.425		
Preoperative MRI-Imaging (no = 1)		0.776		
PVE (no = 1)	2.62 (1.21–5.67)	**0.009**	4.67 (1.31–16.69)	**0.018**
ASA (I/II = 1)		0.341		
Preoperative cholangitis (no = 1)		0,541		
EBD (no = 1)		0.93		
PBD (no = 1)		0.786		
Portal vein infiltration > 180° (no = 1)		0.575		
Arterial infiltration > 180° (no = 1)	2.29 (1.78–4.48)	**0.017**	3.24 (1.26–8.31)	**0.015**
Lobar atrophy (no = 1)		0.687		
sFLR (≤ 40% = 1)		0.109		
Clinical chemistry				
Albumin (≤ 35 g/l = 1)		0.888		
AST (≤ 50 U/l = 1)		0.239		
ALT (≤ 50 U/l = 1)		0.755		
GGT (≤ 400 U/l = 1)		0.593		
Bilirubin (≤ 1 mg/dl = 1)		0.167		
Alkaline phosphatase (≤ 250 U/l = 1)		0.678		
Platelet count (≤ 300/nl = 1)		0.521		
INR (≤ 1 = 1)		0.698		
Hemoglobin (≤ 12 g/dl = 1)		0.08		
CRP, mg/l (≤ 10 mg/l = 1)		0.404		
CA 19–9 U/ml (≤ 250 U/ml = 1)	3.91 (1.71–8.93)	**0.001**	3.2 (1.33–7.69)	**0.009**

Various parameters are associated with non-resectability. Statistically significant *p* values are presented in brackets

*ALT* Alanine aminotransferase, *ASA* American Society of Anesthesiologists classification, *AST* Aspartate aminotransferase, *BMI* Body mass index, *CRP* C-reactive protein, *EBD* Endoscopic biliary drainage, *GGT* Gamma-glutamyltransferase, *INR* International normalized ratio, *PBD* Percutaneous biliary drainage, *PVE* Portal vein embolization

Similar analyses were done excluding patients with R1 resection from the data set. Here, risk factors for non-resectability due to technical reasons were age above 70 years (HR = 3.42, CI 1.38–8.50, *p* = 0.008), PVE (HR = 6.41, CI 1.77–23.15, *p* = 0.005) and arterial infiltration of more than 180° (HR = 7.94, CI 3.16–19.94, *p* < 0.001) in multivariate analysis (Supplementary Table S1). For non-resectability due to oncological and liver function reasons, PVE (HR = 5.18, CI 1.43–18.80, *p* = 0.012), arterial infiltration > 180° (HR = 3.57, CI 1.33–9.62, *p* = 0.012), and CA 19–9 > 250 U/ml (HR = 3.17, CI 1.29–7.81, *p* = 0.012) were independently associated (Supplementary Table S2).

### Survival analysis

**Fig. 1:**
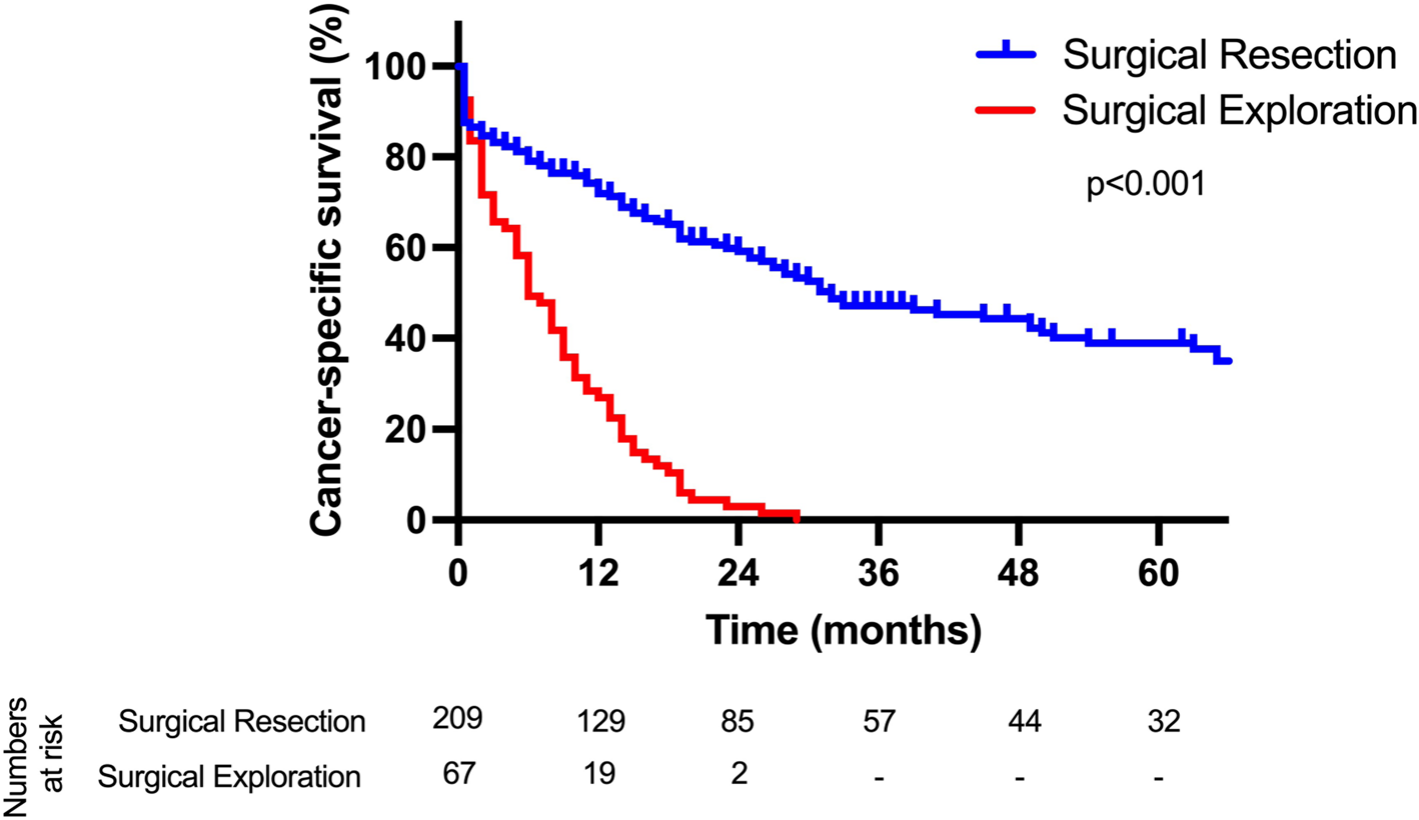
Cancer-specific survival in patients with perihilar cholangiocarcinoma. The median CCS after curative liver resection was 32 months compared to 6 months of surgical exploration without resection. CSS, cancer-specific survival.

To assess the prognostic impact of non-resectability in pCCA, survival analysis was conducted. While the median CSS was 32 months (95%CI 20–44 months) after curative liver resection, a median CCS of 6 months (95%CI 4–8 months) was observed in patients being surgically explored but not resected (*p* = 0.001, Fig. 1) Further, an analysis with respect to resection margin was carried out (Supplementary Figure S1).

## Discussion and conclusion

Curative-intent surgery remains the mainstay of treatment for patients diagnosed with pCCA providing the best long-term prognosis among all available treatment modalities. Assessing surgical resectability upfront is therefore a major goal of the complex preoperative management of these patients. As surgical exploration without actual liver resection results in delayed systemic treatment among other disadvantages for the patient, we here aimed to identify preoperative predictors of non-resectablity in patients with pCCA. Within a large monocentric cohort of resected and surgically explored patients, age, PVE and arterial infiltration diagnosed on preoperative imaging were independent predictors of non-resectability for technical reasons (vascular infiltration without the possibility of reconstruction or resection extend larger than expected) and PVE, arterial infiltration and preoperative CA19-9 major predictors of non-resectability for oncological (distant lymph nodes, liver metastases and peritoneal carcinomatosis) and liver function (intraoperative diagnosis of cirrhosis) reasons. As technical resectability may only be assessed through explorative laparotomy, whereas oncological resectability and liver function could be adequately evaluated via laparoscopic exploration, our data suggests the routine utilization of diagnostic laparoscopy. This is especially relevant in patients displaying high-risk features for futile surgical exploration. Furthermore, survival analysis underlines the oncological impact of non-resectability and emphasizes the need for continuous efforts to improve resectability rates in these patients.

Although identified in separate analyses for oncological and technical reasons for non-resectability, preoperative risk factors such as old age, PVE, arterial infiltration, and elevated preoperative CA19-9 were commonly observed in patients who underwent laparotomy without subsequent resection. 

PVE, as a tool to increase FLR in patients indicated for extended (mostly right-sided) hepatectomy, has been already used for several decades [23]. PVE decreases rates of post-hepatectomy liver failure (PHLF) and mortality and therefore increases resectability in patients with pCCA [24, 25]. Preoperative PVE as a predictor for non-resectability has not been identified in the literature yet. Interestingly, PVE was statistically associated with both non-resectability due to oncological/liver function and technical reasons. A potential explanation might be that patients who underwent PVE had larger and more complex tumors requiring trisectionectomy instead of standard right or left hepatectomy. Thereby, it should be noted that PVE induces a delay in surgery, which increases the chance of tumor progression.

Generally, pCCA with arterial infiltration can be treated in a well-selected patient group with acceptable perioperative complication rates and equivalent oncologic outcomes [26–28]. However, our findings underline arterial infiltration as a still very relevant obstacle in the surgical treatment of pCCA and as a marker of an advanced tumor stage. In a study focusing mostly on patients excluded from surgical therapy/exploration based on preoperative imaging features, arterial involvement as a predictor for non-resectability has also been described by Ruys et al. in 2013 [29]. A current proof-of-principle study in a small cohort showed that hepatic artery involvement in a three-dimensional planning tool based on preoperative CT scans was a risk factor for an R1 or R2 resection [30]. Given those findings, arterial involvement should carefully be evaluated preoperatively [31].

Interestingly, patient age above 70 years was independently associated with non-resectability. Evidence on age as a predictive marker for non-resectability is limited, but patients with pCCA undergoing liver resection with arterial resection/reconstruction tend to be younger in general [26, 32]. Thus, especially in older patients complex vascular resections might be considered intraoperatively as non-resectable due to the associated morbidity. The same accounts for tumors that are intraoperatively assessed as larger than initially expected.

Lastly, elevated CA 19–9 levels showed statistical significance for non-resectability. The value of CA 19–9 as a predictor for resectability was previously demonstrated in single-center analyses based on Asian patients with one study also controlling the prognostic value for hyperbilirubinemia and cholangitis [33, 34]. However, both studies made no differentiation with respect to the reason of irresectability.

Another interesting fact is the irrelevance of Bismuth type IV for non-resectability. This type represents the most frequent in our cohort, implying the generally advanced tumor stage in the group. Our finding in general strengthens an aggressive therapeutical approach also in higher tumor stages.

During the study period, preoperative laparoscopy was not conducted as part of standard clinical management, and upfront laparotomy with consecutive resection in cases showing resectable in preoperative imaging was preferred. The few patients of the unresected cohort who underwent diagnostic laparoscopy (22/109) showed suspicious preoperative imaging findings or were scheduled for the staging procedure for various other reasons. Thus, these patients were excluded from the logistic regressions identifying risk factors for unnecessary laparotomies. However, the other 87 patients which are a notable amount compared to the resected cohort of 209 patients (87/296, 29.4%) during the study period, underwent an unsuccessful laparotomy. It is debatable whether technical non-resectability at the liver hilum or tumor extent of the bile duct can be reliably assessed using diagnostic laparoscopy (37.9%, 33/87). In contrast, peritoneal carcinomatosis, liver metastases or distant nodal metastases as well as impaired quality of the liver parenchyma are easily assessable by means of laparoscopy (59.8%, 52/87). The value of diagnostic laparoscopy was discussed controversially in the past. A systematic review and meta-analysis by Coelen and coworkers included 12 studies with overall 800 patients and showed a pooled sensitivity of 52.2% for diagnostic accuracy of staging laparoscopy in pCCA [35]. However, in the case of peritoneal metastases sensitivity was 80.7%, which seems sufficient to recommend diagnostic laparoscopy. Besides technical limitations, peritoneal carcinomatosis is the main reason for non-resectability in our cohort. In our analysis, we combined oncological reasons for non-resectability and intraoperatively diagnosed low-quality liver parenchyma during explorative laparotomy because both features are assessable by means of diagnostic laparoscopy. Considering the morbidity of almost 45% in the patient group that underwent explorative laparotomy without liver resection and a median hospitalization of 9 days as well as a certain delay in systemic therapy, our results give a strong argument for diagnostic laparoscopy as a staging tool in patients with pCCA. While conventional surgery is currently state-of-the-art for the treatment of pCCA, the integration of minimally invasive robotic liver surgery (MIRLS) could be a valuable tool in the future as new data and studies about this topic indicate [36–38]. Given the first reports of robotic resections in the case of pCCA, it appears also to be feasible to assess resectability within the liver hilum by means of robotic surgery. This would also allow us to clarify technical resectablity in a minimal-invasive manner overcoming the technical limitations of laparoscopy in this regard.

Based on our findings, we propose routine diagnostic laparoscopy to avoid unnecessary laparotomies in patients with high-risk features, e.g., advanced age, preoperative PVE, arterial infiltration, and notable CA 19–9 elevation as these parameters appear strongly to be correlated with irresectability in pCCA patients.

From a theoretical perspective, modern non-invasive diagnostic tools might be considered to omit surgical exploration in some patients. In terms of liver function assessment, the LiMAx test has been used in several studies over the last decade to optimize general patient selection in different indications of liver resection [18, 39, 40]. However, studies focusing on pCCA patients and their specific clinical situation, e.g., after PVE, in the presence of cholangitis and cholestasis are currently not existing. The aforementioned clinical events and complications might interfere with modern liver function tests and therefore reduce the validity of the results for the detection of underlying liver fibrosis or cirrhosis. Based on the given results in other indications, an evaluation of the LiMAx test or other modern function tests appears worthwhile. Also, the role of positron emission tomography (PET), which might detect distant lymph node metastases, is considered controversial in pCCA and CCC in general. In a large systematic review and meta-analysis (2019) a sensitivity of 88.4% and specificity of 69.1% regarding lymph node invasion, and a sensitivity of 85.4% and specificity of 89.7% for distant metastasis was demonstrated for the use of 18F-fluorodeoxyglucose (18FDG) PET for staging in patients with biliary tract cancer. Worse results are reported for the primary tumor with a sensitivity of 91.7% and specificity of only 51.3% for 18FDG-PET [41]. While the PET-Technique might certainly offer a benefit for the intrahepatic subtype of CCA, it is debatable whether pCCA patients (who generally suffer from ongoing cholangitis) also benefit from preoperative PET.

As expected, patients undergoing surgical exploration (both laparoscopically and open) display worse survival than patients proceeding to liver resection (with a median CCS of 6 months compared to 32 months). These results are in accordance with previous studies and underline the superiority of oncological resection compared to palliative care in pCCA [34]. The notable benefit in survival is the main argument for our aggressive approach to the disease with a large amount of trisectionectomies and vessel reconstructions in our cohort. It is also a good argument to conduct surgery in the elderly after careful case-by-case evaluation of the individual fitness of the patient.

Fairly, some potential limitations must be mentioned according to this study. As a single-centered study, all results reflect the authors’ individual therapeutic approach to pCCA. Our strategies comprise an aggressive approach to the disease with vessel resection and reconstruction on demand. Thus, a subset of patients might not have been subjected to surgical exploration in the setting of a more conservative approach to surgical treatment of pCCA. Also, does our monocentric data warrant further validation by independent data sets? Further, the retrospective nature of the study does compromise the generalizability, does not allow accuracy as controlled prospective studies, and might introduce undetected bias. Admittedly, the presented data is based on an inclusion period of more than 10 years in which the role and technical possibilities of laparoscopy have substantially changed.

Considering the limitations, we identified advanced Age, PVE, and arterial infiltration in the preoperative imaging as independent predictors for non-resectability due to technical reasons in the setting of explorative laparotomy. PVE, arterial infiltration, and elevated CA19-9 are independent predictors for non-resectability due to oncological/liver function reasons. Critical evaluation and assessment of these mostly easily available parameters are recommended for better therapeutical pathways. Thus, diagnostic laparoscopy, especially in these high-risk situations, should be used to reduce the amount of explorative laparotomies without subsequent liver resection. Finally, our findings should further be assessed in future multicentric and prospective studies.

### Supplementary Information

**Additional file 1: Supplementary Figure S1.** Cancer-specific survival in patients with perihilar cholangiocarcinoma. The median CCS after R0 resection was 41 months compared to 16 months after R1 resection and 6 months of surgical exploration without resection. CSS, cancer-specific survival.

**Additional file 2: Supplementary Table S1.** Logistic regression of preoperative parameters for non-resectability due to technical reasons (Patients with R1-situation excluded).

**Additional file 3: Supplementary Table S2.** Logistic regression of preoperative parameters for non-resectability due to oncological reasons/liver function (Patients with R1-situation excluded).

## Data Availability

No datasets were generated or analysed during the current study.

## Abbreviations

ALPPS: Associating liver partition and portal vein ligation for staged hepatectomy
ALT: Alanine aminotransferase
AP: Alkaline phosphatase
ASA: American Society of Anesthesiologists
AST: Aspartate aminotransferase
BMI: Body mass index
CA 19-9: Carbohydrate antigen 19–9
CCA: Cholangiocellular carcinoma
CI: Confidence interval
CRP: C reactive protein
CSS: Cancer-specific survival
CT: Computed tomography
CUSA: Cavitron Ultrasonic Surgical Aspirator
EBD: Endoscopic biliary drainage
ERCP: Endoscopic retrograde cholangiopancreatography
FFP: Fresh frozen plasma
FLR: Future liver remnant
GGT: Gamma-glutamyltransferase
INR: International normalized ratio
LiMAx: Maximum liver function capacity
MIRLS: Minimally invasive robotic liver surgery
MRCP: Magnetic resonance cholangiopancreatography
MRI: Magnetic resonance imaging
PBD: Percutaneous biliary drainage
PET-CT: Positron emission tomography–computed tomography
PVE: Portal vein embolization
PHLF: Post hepatectomy liver failure
pCCA: Perihilar cholangiocarcinoma
RWTH: Rheinisch-Westfälische Technische Hochschule
