# Supplementary figures and images for: Preoperative predictors for non-resectability in perihilar cholangiocarcinoma
Source: World J Surg Oncol. 2024 Feb 7;22:48. doi: 10.1186/s12957-024-03329-1 (PMC10851609; doi:10.1186/s12957-024-03329-1)

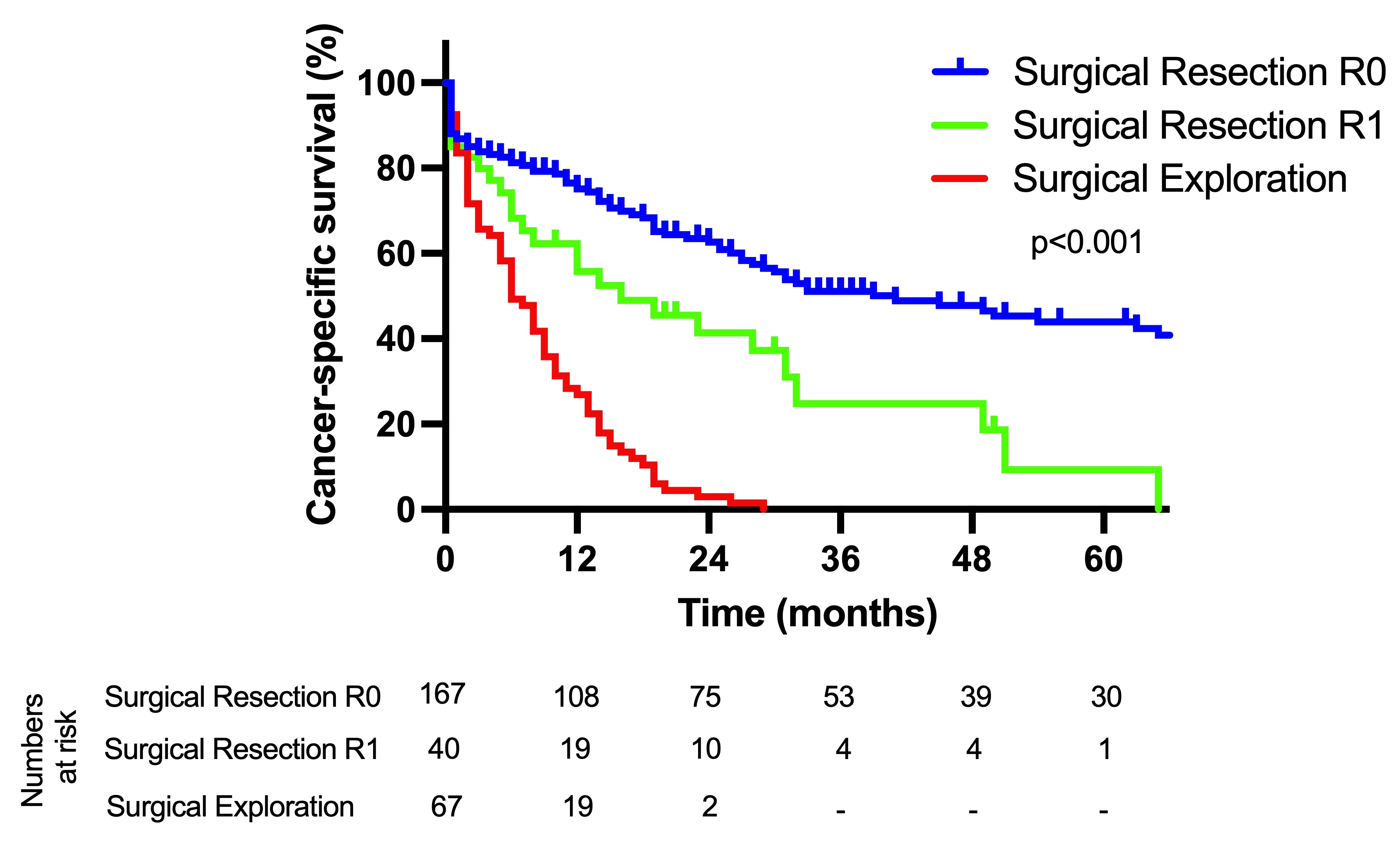

Supplement: Supplementary file 1 — Additional file 1: Supplementary Figure S1. Cancer-specific survival in patients with perihilar cholangiocarcinoma. The median CCS after R0 resection was 41 months compared to 16 months after R1 resection and 6 months of surgical exploration without resection. CSS, cancer-specific survival. [file 12957_2024_3329_MOESM1_ESM.jpg]
