# Supplementary material for: Preoperative predictors for non-resectability in perihilar cholangiocarcinoma
Source: World J Surg Oncol. 2024 Feb 7;22:48. doi: 10.1186/s12957-024-03329-1 (PMC10851609; doi:10.1186/s12957-024-03329-1)
Supplement: Supplementary file 2 — Additional file 2: Supplementary Table S1. Logistic regression of preoperative parameters for non-resectability due to technical reasons (Patients with R1-situation excluded). [file 12957_2024_3329_MOESM2_ESM.docx]

**Supplementary Table 1:** Logistic regression of preoperative parameters for non-resectability due to technical reasons (Patients with R1-situation excluded)

|  | **Univariate analysis** | |  | **Multivariate analysis** | |
| --- | --- | --- | --- | --- | --- |
|  | **HR (95% CI)** | ***P* value** |  | **HR (95% CI)** | ***P* value** |
| **Demographics** |  |  |  |  |  |
| Sex (male=1) |  | 0.083 |  |  |  |
| Age (≤ 70 years=1) | 2.18 (1.02 – 4.66) | **0.046** |  | 3.42 (1.38 – 8.50) | **0.008** |
| BMI (≤ 25 kg/m^2^=1) |  | 0.605 |  |  |  |
| Bismuth type (I/II=1) | 2.22 (1.02 – 4.79) | **0.044** |  |  | 0.670 |
| Neoadjuvant therapy (no=1) |  | 0.667 |  |  |  |
| Preoperative MR-Imaging (no=1) |  | 0.622 |  |  |  |
| PVE (no=1) | 5.79 (1.7 – 19.77) | **0.005** |  | 6.41 (1.77 – 23.15) | **0.005** |
| ASA (I/II=1) |  | 0.479 |  |  |  |
| Preoperative cholangitis (no=1) |  | 0.800 |  |  |  |
| EBD (no=1) |  | 0.950 |  |  |  |
| PBD (no=1) |  | 0.358 |  |  |  |
| Portal vein infiltration > 180° (no=1) |  | 0.121 |  |  |  |
| Arterial infiltration > 180° (no=1) | 4.91 (2.24 – 10.75) | **<0.001** |  | 7.94 (3.16 – 19.94) | **<0.001** |
| Lobar atrophy (no=1) |  | 0.672 |  |  |  |
| sFLR (≤ 40%=1) |  | 0.371 |  |  |  |
| **Clinical chemistry** |  |  |  |  |  |
| Albumin (≤ 35 g/l=1) |  | 0.257 |  |  |  |
| AST (≤ 50 U/l=1) |  | 0.561 |  |  |  |
| ALT (≤ 50 U/l=1) |  | 0.297 |  |  |  |
| GGT (≤ 400 U/l=1) |  | 0.271 |  |  |  |
| Bilirubin (≤ 1 mg/dl=1) |  | 0.891 |  |  |  |
| Alkaline phosphatase (≤ 250 U/l=1) |  | 0.480 |  |  |  |
| Platelet count (≤ 300 /nl=1) |  | 0.413 |  |  |  |
| INR (≤ 1=1) |  | 0.355 |  |  |  |
| Hemoglobin (≤ 12 g/dl=1) |  | 0.586 |  |  |  |
| CRP, mg/l (≤ 10 mg/l=1) |  | 0.611 |  |  |  |
| CA 19-9, U/ml (≤ 250 U/ml=1) |  | 0.601 |  |  |  |

*Various parameters are associated with non-resectability. ALT, alanine aminotransferase; ASA, American society of anesthesiologists classification; AST, aspartate aminotransferase; BMI, body mass index; CRP, c-reactive protein; EBD, endoscopic biliary drainage; GGT, gamma glutamyltransferase; INR, international normalized ratio; PBD, percutaneous biliary drainage; PVE, portal vein embolization. Statistically significant p-values are presented in brackets.*
