# Supplementary material for: Preoperative predictors for non-resectability in perihilar cholangiocarcinoma
Source: World J Surg Oncol. 2024 Feb 7;22:48. doi: 10.1186/s12957-024-03329-1 (PMC10851609; doi:10.1186/s12957-024-03329-1)
Supplement: Supplementary file 3 — Additional file 3: Supplementary Table S2. Logistic regression of preoperative parameters for non-resectability due to oncological reasons/liver function (Patients with R1-situation excluded). [file 12957_2024_3329_MOESM3_ESM.docx]

**Supplementary Table 2:** Logistic regression of preoperative parameters for non-resectability due to oncological reasons/liver function (Patients with R1-situation excluded)

|  | **Univariate analysis** | |  | **Multivariate analysis** | |
| --- | --- | --- | --- | --- | --- |
|  | **HR (95% CI)** | ***P* value** |  | **HR (95% CI)** | ***P* value** |
| **Demographics** |  |  |  |  |  |
| Sex (male=1) |  | 0.431 |  |  |  |
| Age (≤ 70 years=1) |  | 0.398 |  |  |  |
| BMI (≤ 25 kg/m^2^=1) |  | 0.926 |  |  |  |
| Bismuth type (I/II=1) |  | 0.275 |  |  |  |
| Neoadjuvant therapy (no=1) |  | 0.415 |  |  |  |
| Preoperative MRI-Imgaging (no=1) |  | 0.720 |  |  |  |
| PVE (no=1) | 2.77 (1.27 – 6.06) | **0.006** |  | 5.18 (1.43 – 18.80) | **0.012** |
| ASA (I/II=1) |  | 0.436 |  |  |  |
| Preoperative cholangitis (no=1) |  | 0.594 |  |  |  |
| EBD (no=1) |  | 0.884 |  |  |  |
| PBD (no=1) |  | 0.730 |  |  |  |
| Portal vein infiltration > 180° (no=1) |  | 0.508 |  |  |  |
| Arterial infiltration > 180° (no=1) | 2.17 (1.09 – 4.30) | **0.027** |  | 3.57 (1.33 – 9.62) | **0.012** |
| Lobar atrophy (no=1) |  | 0.813 |  |  |  |
| sFLR (≤ 40%=1) |  | 0.090 |  |  |  |
| **Clinical chemistry** |  |  |  |  |  |
| Albumin (≤ 35 g/l=1) |  | 0.902 |  |  |  |
| AST (≤ 50 U/l=1) |  | 0.187 |  |  |  |
| ALT (≤ 50 U/l=1) |  | 0.711 |  |  |  |
| GGT (≤ 400 U/l=1) |  | 0.603 |  |  |  |
| Bilirubin (≤ 1 mg/dl=1) |  | 0.196 |  |  |  |
| Alkaline phosphatase (≤ 250 U/l=1) |  | 0.734 |  |  |  |
| Platelet count (≤ 300 /nl=1) |  | 0.609 |  |  |  |
| INR (≤ 1=1) |  | 0.677 |  |  |  |
| Hemoglobin (≤ 12 g/dl=1) |  | 0.189 |  |  |  |
| CRP, mg/l (≤ 10 mg/l=1) |  | 0.339 |  |  |  |
| CA 19-9 U/ml (≤ 250 U/ml=1) | 3.69 (1.59 – 8.57) | **0.002** |  | 3.17 (1.29 – 7.81) | **0.012** |

*Various parameters are associated with non-resectability. ALT, alanine aminotransferase; ASA, American society of anesthesiologists classification; AST, aspartate aminotransferase; BMI, body mass index; CRP, c-reactive protein; EBD, endoscopic biliary drainage; GGT, gamma glutamyltransferase; INR, international normalized ratio; PBD, percutaneous biliary drainage; PVE, portal vein embolization. Statistically significant p-values are presented in brackets.*
